# Exacerbation of Spinal Stenosis Symptoms Following Neuraxial Anesthesia in an Achondroplastic Cesarean Section

**DOI:** 10.7759/cureus.45170

**Published:** 2023-09-13

**Authors:** Sean P Renfree, Jack Haglin, Joseph C Brinkman, Andrew Chung

**Affiliations:** 1 Orthopedic Surgery, University of Arizona College of Medicine, Tucson, USA; 2 Orthopedic Surgery, Mayo Clinic, Scottsdale, USA; 3 Orthopedic Surgery, Banner Health, Phoenix, USA

**Keywords:** lumbar spinal stenosis (lss), cesarean section (cs), general anesthesia, neuraxial anesthesia complications, homozygous achondroplasia

## Abstract

We report the case of an achondroplastic female who presented with acute neurologic decline following epidural anesthesia for an elective cesarean section. Achondroplasia presents unique anatomical challenges to anesthesiologists in perioperative management, and cesarean sections are standard for achondroplastic pregnancies. High rates of spinal stenosis and lumbar radiculopathy in this patient population make administration of epidural analgesia technically challenging and may increase the risk of neurologic injury. Ultrasound is an effective means of administering epidural anesthesia for most patients; however, its utility is user-dependent and more challenging for those with obesity and abnormal spinal anatomy, both of which are common in achondroplasia. Cephalic and thoracic anatomical features in achondroplasia can also make general anesthesia challenging. Therefore, preoperative imaging may help guide preoperative planning based on patient anatomy and individual risk factors to reduce the risks of complications in this patient population. This report includes details from the patient’s prenatal care, cesarean section, and 18 months of follow-up.

## Introduction

Achondroplasia is the most common form of skeletal dysplasia and cause of disproportionate short stature [1]. This congenital condition develops from impaired endochondral ossification from an activating mutation of fibroblast growth factor receptor 3, which causes impaired longitudinal bone growth and commonly leads to spinal pathology [2]. Cesarean delivery is standard in pregnant mothers with achondroplasia due to uniform narrowing of the pelvis and cephalopelvic disproportion from macrocephaly in an achondroplastic fetus [3]. The skeletal morphology in achondroplastic dwarfs (ADs) presents unique anatomical challenges to anesthesiologists in perioperative management. For example, ADs have some degree of foramen magnum stenosis, kyphoscoliosis, lumbar lordosis, lumbar spinal stenosis, and limited neck extension [2,4,5]. Therefore, all achondroplastic pregnancies should ideally be evaluated by an anesthesiologist as part of prenatal care. Epidural analgesia in ADs has been shown to be effective in a growing number of case reports with limited follow-up periods [6-10]. However, high rates of spinal stenosis and lumbar radiculopathy make administration of epidural analgesia technically challenging and may increase the risk of neurologic injury [11]. We present the management of an achondroplastic patient who received epidural analgesia for an elective cesarean section and developed an exacerbation of neurological symptoms later requiring lumbar decompression.

## Case presentation

A 29-year-old gravida 2, para 1 AD underwent a planned cesarean section at 38 weeks’ gestational age. The patient’s medical history included a previous cholecystectomy under general anesthesia, open reduction internal fixation for an olecranon fracture under general anesthesia, tonsillectomy under general anesthesia, and six tympanostomy-related procedures all under general anesthesia. The patient underwent all procedures more than seven years prior to presentation and without complications. She has a history of intermittent, mild back pain and right unilateral radicular pain without weakness beginning at 18 years of age. She underwent an MRI within a few months of the onset of her first symptoms while under the care of her pediatric orthopedists and was diagnosed with spinal stenosis. Her intermittent symptoms from spinal stenosis remained consistent after her original diagnosis and through her first pregnancy.

Her first pregnancy occurred two years prior to presentation, and she delivered a healthy achondroplasia female at 39 weeks’ gestation via planned cesarean section. Anesthesia was given via spinal epidural but with two failed attempts at different locations. The third attempt achieved successful placement but resulted in hypotension requiring vasopressors. However, the remainder of the delivery through discharge was uneventful. She denied any change in her neurological symptoms related to her stenosis during both peripartum and postpartum periods of her first pregnancy.

The patient’s second pregnancy was complicated by COVID-19 infection during the first trimester, anemia (hemoglobin of 10.8 g/dL), and polyhydramnios above the 95th percentile, all occurring after 30 weeks’ gestation. During the fourth month of pregnancy, she began experiencing persistent, moderate back pain, and unilateral radiculopathy. However, by the sixth month, her pain increased in severity, and she began experiencing bilateral radiculopathy for the first time. Her musculoskeletal examination was noted for persistent right-sided lower back pain and reduced strength of her right lower extremity, particularly weak with knee flexion, knee extension, dorsiflexion, and plantarflexion (all 4/5). She also endorsed bilateral radiculopathy extending to the posterolateral ankles exacerbated with forward hip flexion at standing position. She was placed on bed rest for the remainder of the pregnancy due to intermittent hypotension and pain with ambulation. Her prenatal care and cesarean section delivery were managed by the same provider as her previous pregnancy.

The patient presented to the labor floor for planned cesarean section at 38 weeks’ gestational age. She was 127 cm tall and weighed 66 kg with a body mass index of 40.8 kg/m2. An examination revealed a Mallampati III airway with a 5-cm thyromandibular distance with mild kyphoscoliotic deformity observed in the thoracolumbar spine. Cardiorespiratory examination was unremarkable. The patient was deemed an American Society of Anesthesiologists class III physical status.

A combined spinal-epidural anesthesia procedure was performed with the patient in the sitting position. A 25-gauge Pencan® Pencil Point Spinal Needle was inserted into the L3-L4 interspace. A mixture of fentanyl 20 µg and morphine 150 µg was administered into the spinal space, followed by 0.75% hyperbaric bupivacaine 1 mL. The sensory level was assessed, achieving a T4-level sensory blockade. Three minutes following administration of the epidural, the patient became hypotensive, and thus intravenous phenylephrine 200 µg and atropine 0.4 mg were promptly administered, stabilizing blood pressure. The cesarean section started 10 minutes after administering the neuraxial anesthesia. An additional 500 µg of phenylephrine was administered during the procedure to maintain appropriate blood pressure. After a Pfannenstiel skin incision cesarean section, a healthy achondroplastic female was delivered. The patient received intravenous oxytocin for post-operative pain control. The entire surgery lasted less than an hour. The remainder of the surgical and immediate postoperative recovery of the patient was unremarkable without a significant change in neurologic symptoms. She was discharged two days later.

**Figure 1:**
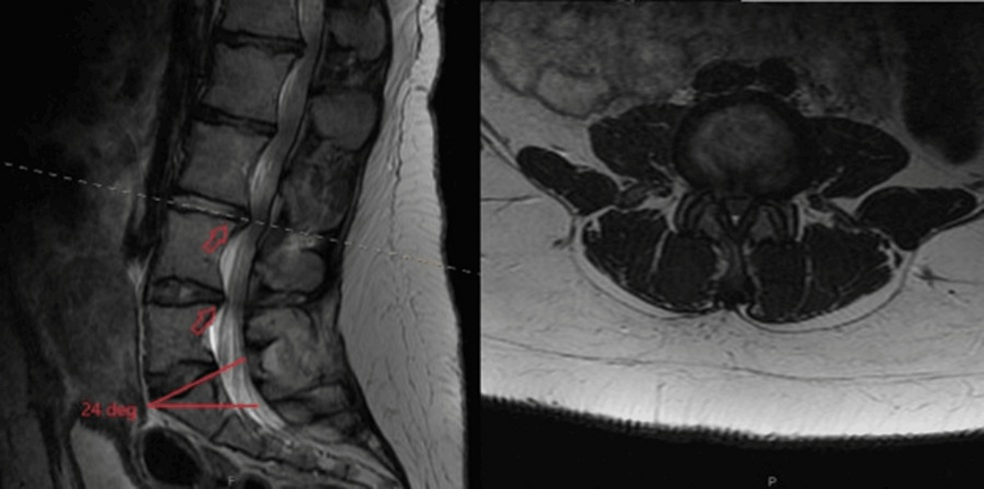
Preoperative T2-weighted Lumbar MRI. Left image (sagittal view): Broad disc bulges (red arrows) at L3/L4 and resulting descending lumbar nerve root compression; Ferguson angle is 24 degrees. Right image (axial view of L3/L4): severe canal stenosis and facet arthropathy with advanced lateral recess stenosis can be seen.

**Figure 2:**
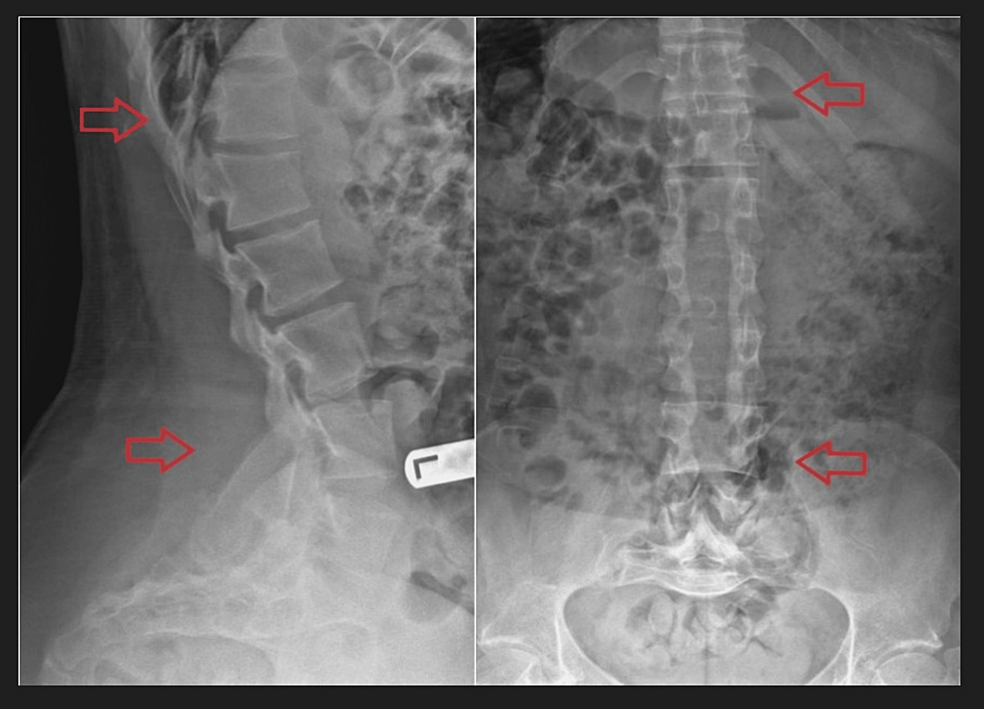
Postoperative X-ray following T12-L5 laminectomy with bilateral partial facetectomy. Left image (lateral view): red arrows indicating theterminal vertebrae (T12/L5) of the involved laminectomy. Right image (anteroposterior view): red arrows indicating theterminal vertebrae (T12/L5) of the involved laminectomy.

The patient continued to experience persistent back pain and bilateral radiculopathy; however, she no longer received relief with rest and developed profound lower extremity weakness over three weeks. Four weeks after her cesarean section, her musculoskeletal examination revealed 2/5 strength with right knee flexion, knee extension, dorsiflexion, and plantarflexion compared to 3/5 strength with the same tests of the contralateral extremity. At this point, she was prescribed physical therapy, but within a week, she could no longer reliably travel to therapy or perform stationary exercises. She only received temporary pain relief sitting in a reclining chair, including for sleep. At this time, she was concerned about her ability to care for her infant due to her significant limitations. She subsequently underwent an MRI, which revealed moderate-to-severe diffuse lumbar spinal canal stenosis (greatest from L1-L2 through L4-L5), with advanced lateral recess stenosis, diffuse lumbar foraminal narrowing from facet arthropathy, and lateral disc disease (Figure 1). Given the diffuse multilevel lumbar stenosis on MRI, she promptly underwent T12-L5 laminectomy with bilateral partial facetectomy for central canal and bilateral lateral recess stenosis without complications (Figure 2). This procedure was performed seven weeks after her cesarean section. She was discharged six days after the laminectomy to a rehabilitation center for seven days and required the assistance of a walker for four weeks. Over the ensuing months, she regained full strength, and 18 months postoperatively she continued to deny any kind of numbness or paranesthesia.

## Discussion

Cesarean delivery is necessitated in achondroplastic pregnancies due to uniform narrowing of the pelvis and cephalopelvic disproportion from macrocephaly in an achondroplastic fetus. Consequently, all achondroplastic pregnancies should be evaluated by an anesthesiologist as part of prenatal care to determine optimal anesthesia management. There are a number of important patient characteristics to consider for those with achondroplasia.

Spinal pathology of the lumbar region in achondroplasia makes epidural anesthesia challenging. ADs have diminished caliber of the spinal canal with features including short and thick pedicles, hypertrophy of superior and inferior articular facets, prominent bulging of intervertebral discs, and varying degrees of thoracic kyphosis and lumbar lordosis [2]. Additionally, the spinal canal tapers caudally, decreasing the interpedicular distance in the lumbar region with a great degree of variation in cross-sectional area in the lower lumbar region regardless of symptomology [4,11]. These anatomic features make neuraxial access difficult and epidural anesthetic spread unpredictable, with an increased risk of dural puncture and total spinal anesthesia resulting in hypotension [4,5]. Because uterine blood flow and fetal oxygenation are directly related to maternal arterial pressure, hypotension is highly problematic and must be addressed immediately [12]. This scenario occurred in our patient and, fortunately, was quickly addressed with vasopressors. Moreover, there is a great degree of variation in the amount of anesthetic required to achieve acceptable anesthesia, which has been described in detail in previous case reports [5,6]. Despite the common spinal pathology in achondroplasia, symptomology of spinal stenosis may vary, and many patients remain asymptomatic into the third decade of life [4,11,13]. Therefore, clinical assessment of the severity of spinal stenosis in achondroplasia may be difficult in pregnant women without supplemental imaging.

Cephalic and thoracic anatomy also makes general anesthesia challenging. Achondroplastic dwarfs commonly suffer from micrognathia, macroglossia, laryngotracheal stenosis, foramen magnum stenosis, thoracolumbar kyphoscoliosis, and limited neck extension, making intubation difficult [2,4,5]. Additionally, a small, narrow chest cage with reduced symphysis pubis to xiphoid distance reduces respiratory capacity [14]. The achondroplastic thorax is smaller and becomes impacted sooner from a narrower pelvis, causing upward displacement of the uterus. As a result, patients can experience respiratory insufficiency in the third trimester, making general anesthesia difficult [15]. In the general population, general anesthesia is considered a second-line approach for cesarean deliveries given its increased odds of developing anesthesia complications, surgical site infection, and venous thromboembolism [16]. However, no studies have been conducted to assess odds for complications in ADs.

Achondroplastic pregnancies pose their own unique challenges, with very little literature available on anesthesia management for cesarean sections. Due to the relative rarity of achondroplasia and a wide range of anatomical risk factors, definitive guidelines do not exist for perioperative management for cesarean sections. A panel of 13 multidisciplinary international experts recommended appropriate screening for risk factors to consider for regional anesthesia. Unfortunately, specific recommendations were not provided regarding the assessment of these risk factors [15]. Consequently, clinicians are faced with the challenge of assessing risk factors on an individual basis in a patient population that may be unfamiliar to them. Given that ADs have considerable risk factors for both spinal epidural and general anesthesia management, preoperative imaging seems appropriate to guide shared decision-making. It is our view that a one-time lumbar MRI may help assess the severity of lumbar spinal stenosis, which has been shown to be highly variable among ADs [11]. This strategy will allow clinicians to optimize patient selection for epidural anesthesia and plan for epidural placement under ultrasound guidance for appropriate candidates.

MRI may be warranted for achondroplastic adults undergoing elective procedures, even for those who may be asymptomatic at presentation. A previous MRI study on ADs demonstrated that there is a poor correlation between stenosis and symptomology at the lower lumbar levels (L4/L5) [11]. While ultrasound is an effective means of administering epidural anesthesia for most patients, its utility is user-dependent and more challenging for those with obesity and abnormal spinal anatomy [17], both of which are common in achondroplasia. A previous case report noted difficulty with ultrasound in identifying the dura mater and determining the depth of the epidural space highlighting this issue [7]. Therefore, a preoperative MRI provides adequate clarity for a team to generate a targeted plan to supplement ultrasound-guided epidural placement. This preoperative strategy has been applied in two prior case reports with success, with both reports acknowledging its utility in identifying variations in spinal stenosis throughout the lumbar spine and assessing the interspinous space [8,9]. Porter and Mendonca justified the use of MRI to ensure successful epidural administration as part of preoperative planning due to pregnancy-related depressed respiratory function that would have necessitated postoperative ventilatory support [9]. Similarly, Fiol et al. obtained an MRI for preoperative planning to improve the chances of a successful block for a patient who insisted on receiving epidural anesthesia early in the pregnancy [8]. Though kyphoscoliotic deformity of the thoracolumbar spine was observed in both cases, neither reported a history of significant neurologic symptoms. Both reports acknowledged that MRI imaging facilitated identification of the conus medullaris and areas of interspace narrowing, thereby guiding epidural anesthetic administration. Thus, MRI of the spine may guide clinicians in making an informed decision regarding anesthetic management for achondroplastic patients.

While it is purely speculative to assume that our patient would have benefited from MRI, this particular case encourages the discussion regarding appropriate preoperative imaging for ADs. Patients in the general population with preexisting spinal canal pathology, including spinal stenosis and lumbar disk disease, are often considered poor candidates for neuraxial blockade, and thus may receive general anesthesia [18]. However, ADs commonly share prominent risk factors for undergoing general anesthesia as previously mentioned; therefore, epidural anesthesia may be necessary in these particularly high-risk patients. In such high-risk patients, MRI seems prudent to minimize the risk of complications during epidural administration. Nevertheless, imaging may still be indicated for all ADs as many remain asymptomatic despite severe stenosis [11]. We also believe that this approach can be applied to elective procedures that commonly utilize epidural anesthesia.

## Conclusions

To our knowledge, this is the first case report to describe the worsening of neurological symptoms from spinal stenosis following epidural anesthesia in an AD. Previous reports have provided evidence that epidural anesthesia can be successfully utilized in ADs. However, case reporting is problematic as this format rarely provides long-term follow-up to assess patients who go on to develop neurological sequela following epidural analgesia. Most case reports recount the procedural aspect of the epidural and cannot provide insight into adverse events after discharge. We were fortunate to report on this case because our patient presented for surgical evaluation to address worsening symptoms and included 18 months of follow-up. There is a need for case-control studies to provide further clarity on such issues faced by this patient population. Though case-control studies have been historically difficult to conduct for conditions with small patient populations, the invention of large-scale national databases may be able to provide further insight. Nonetheless, given the number of anatomical challenges posed by achondroplasia, management should be handled by high-risk multidisciplinary care teams. A comprehensive preoperative assessment, effective communication, and multidisciplinary team approach play a critical role in the perioperative management of pregnant patients with achondroplasia.
